# *Ruta graveolens* and rutin, as its major compound: investigating their effect on spatial memory and passive avoidance memory in rats

**DOI:** 10.1080/13880209.2020.1762669

**Published:** 2020-05-20

**Authors:** Shirin Asgharian, Mohammad Reza Hojjati, Mohsen Ahrari, Elham Bijad, Fatemeh Deris, Zahra Lorigooini

**Affiliations:** a Medical Plants Research Center, Basic Health Sciences Institute, Shahrekord University of Medical Sciences, Shahrekord, Iran; b Department of Physiology, Medical Faculty, Shahrekord University of Medical Sciences, Shahrekord, Iran; c Department of Epidemiology and Biostatistics, School of Health, Shahrekord University of Medical Sciences, Shahrekord, Iran

**Keywords:** Learning tests, malondialdehyde, antioxidants

## Abstract

**Context:**

There are numerous pharmacological activities for *Ruta graveolens* and its bioactive constituent, rutin, on learning and memory.

**Objective:**

This study aimed to examine the effect of *R. graveolens* and rutin on memory in rats.

**Materials and methods:**

In this study animals were treated with the hydroalcholic extract of *R. graveolens* and rutin by IP injection for 10 days. Behavioural and biochemical tests as well as HPLC analysis and antioxidant activity of extract have been evaluated.

**Results:**

*R. graveolens* extract and rutin significantly increased learning and improved spatial memory, as well as secondary latency; moreover, there were significant increases in the serum and brain antioxidant capacity as well as the level of TBARS in serum and brain tissues. Results also showed that *R. graveolens* has significant DPPH radical scavenging effect (IC_50_: 159.17 ± 1.56 μg/mL). The HPLC analysis of extract showed that caffeic acid (19.92 ± 0.01), rutin (40.15 ± 0.01), and apigenin (0.84 ± 0.01) mg/g of dry extract are the main components of the extract.

**Discussion and conclusion:**

Regarding the effects of *R*. *graveolens* extract and rutin on animal brain cells, memory function, and learning, additional studies, including clinical trials, might be beneficial in producing natural supplementary drugs from this herb.

## Introduction

Learning and memory are the hallmarks of human beings. Learning and memory are among the highest functional levels of the central nervous system (CNS) (Spencer et al. 2009). Memory is a biological adaptation that enables a living organism to use past experiences to regulate its behaviour against environmental changes. Memory is assumed to be the result of long-term changes induced by learning in the relationship between neurons (Holroyd and Shepherd 2001). The spatial memory and long-term memory are constantly forming in the hippocampus (Diba et al. 2018). Since learning and memory are important issues in science and technology advancement and the number of people with learning and memory disorders is increasing, using herbal drugs in the treatment of forgetfulness and enhancing memory has been a topic of interest for many researchers (Izadpanah et al. 2017). These herbs have been used since ancient times and they rarely lead to side effects (Jamshidi-Kia et al. 2018). There are many medicinal herbs effective in enhancing memory and learning. *Ruta graveolens* L. (Rutaceae), which has significant therapeutic properties, is one of the medicinal herbs used in traditional medicine in Iran and some other countries (Shojaii et al. 2016). This herb is referred to as panacea due to its high therapeutic value include the treatment of anticular, ear, pharynx, neurological, pulmonary, digestive, renal, gynecological diseases, parasites excretion, pain relief, and treatment of spasm and inflammation **(**Asgarpanah and Khoshkam 2012; Ratheesh et al. 2013; Baharvand-Ahmadi et al. 2015; Javadi and Emami 2015). There are more than 120 compounds of different types of flavonoid glycosides, quinoline alkaloids, coumarins, lignins, and flavonoids as the active ingredients of *R*. *graveolens* (Stashenko et al. 2000; Gentile et al. 2015). Among these flavonoids, rutin has been a topic of interest for researchers (Adsersen et al. 2006). Also, as a major compound of *R*. *graveolens*, rutin enhances memory retrieval and has pharmacological activities, including anti-allergic, anti-inflammatory, anticancer, antiviral, and antimycotic properties (Jianxiong et al. 2008). Therefore, the present study was conducted to investigate and compare the effect of hydroalcholic extract of *R*. *graveolens* and rutin on spatial memory and passive avoidance memory in rats.

## Materials and methods

### Extraction

Fresh *R. graveolens* was purchased from local market on April 2016. Moreover, after identifying the herb by an expert botanist (Shirmardi, Hamzeh Ali, PhD., Research Centre of Agriculture and Natural Resources, P.O. Box 415, Shahrekord, Iran); it was deposited to the Herbarium Unit of Shahrekord University of Medical Sciences (Herbarium No. 152). To extract the dried and pulverized sample, ethanol 70% was used. The obtained extract was filtered and concentrated in a rotary vacuum evaporator after 72 h. Furthermore, the concentrated extract was incubated at 37 °C for final drying (Pandey et al. 2012).

### Determining the antioxidant capacity of the extract

After preparation and incubation of the extract stock solution and DPPH (diphenyl picryl hydrazine) at room temperature and in the dark for 15 min, the absorbance of the samples was recorded at 517 nm wavelength. Methanol was used as blank and methanol and DPPH were used as control. By measuring the quantity of free radicals inhibited by the extract, the antioxidant activity was reported to be IC_50_ **(**Ghasemi et al. 2009).

### Determination of total phenolic and flavonoid content

Total phenolic and flavonoid content of the extract was determined using Folin-Ciocalteu reagent and the aluminium chloride colorimetric method, respectively. To achieve this purpose, total phenolic and flavonoid content was calculated by the calibration curve, and the results were expressed as mg gallic acid equivalent (GAE) and rutin equivalent/g of the extract dry weight, respectively (Medini et al. 2014).

### Standardisation and analysis of flavonoid and phenolic compounds by HPLC

HPLC was used for qualitative analysis of the phenolic acid and flavonoids (Knauer, Germany). The system was equipped with a C18 column (5 µm particle size, i.d. 250 mm × 4.6 mm) and UV–visible detector (PDA Detector 2800). The binary mobile phase, including solvent A (methanol and 0.05% trifluoroacetic acid) and solvent B (deionized water and 0.05% trifluoroacetic acid) was eluted as follows to wash and equilibrate the column: 0–10 min, 20% solvent A and 80% solvent B; 10–40 min, 30% solvent A and 70% solvent B; 40–45 min, 60% solvent A and 40% solvent B; 45–50 min, 80% solvent A and 20% solvent B; and 50–55 min, 100% solvent A and 0% solvent B. The flow rate was 0.5 mL/min and the injection volume was 20 µL. Detection was performed by scanning at 190–800 nm wavelength and reading the absorbance at 280–372 nm wavelength. Phenolic acid and flavonoids were identified by comparing their UV spectra and retention times to the analytical standards. Also, a calibration curve equation was used to calculate the content of each flavonoid and phenolic acid, and the results were expressed as µg/mg of the extract dry weight.

### Laboratory animals

The study protocol was designed according to the ‘Guide for the Care and Use of Laboratory Animals’, approved by the Research Ethics Committee of Shahrekord University of Medical Sciences (IR.SKUMS.REC.1394.241). 40 male Wistar rats weighing 250–300 g were purchased for this study from Pasteur Institution (Tehran, Iran). Rats were kept at the temperature of 21 ± 2 °C, under 12 h light/dark cycle with free access to similar food and water. The rats were assigned to 8 groups (*n* = 5), including group 1: receiving normal saline (1 mg/kg), groups 2, 3, and 4: intraperitoneally receiving *R. graveolens* extract with concentrations of 30, 100 and 300 mg/kg, respectively (Sailani and Moeini 2007), and group 5: receiving rutin (10 mg/kg) (Nassiri-Asl et al. 2010).

### Morris water maze test

Each rat was given 60 min to find the platform in Morris water maze test. If a rat could not find the platform, it was guided to the platform. The rats were allowed to rest for 30 s between each two trials to explore the environment. Also, rats were taken out from water for 10 min between two blocks, and those were allowed to rest in the cage. Each rat was trained 4 times per day for 4 days, and the test was repeated without the platform on day 5 (probe day) (Tarnawski et al. 2006).

### Shuttle box test

The shuttle box test lasted 4 days for each rat. During the first and second days, each rat was left in the box to acclimatize to it. Moreover, an acquisition test was conducted on day 3. The rats were separately placed in the bright chamber of the shuttle box for this purpose. After a 2 min acclimatisation period, guillotine door was opened and after the rat got into the dark chamber, it was closed, and then an electrical shock (1 mA, for a second) was exerted to the rat, such that it only paddled. In this test, the length of primary latency in entering the dark chamber on the day 3 and the length of latency during passing on the day 4 were recorded (Rabiei et al. 2014).

### Measuring serum and the brain antioxidant capacity

Three solutions were used in order to measure serum and brain antioxidant capacity: 1. Buffer (1.55 mL sodium acetate, 8 mL concentrated acetic acid reaching 500 mL by adding distilled water), 2. Ferrous chloride solution (270 mg FeCl_3_ (6 H_2_O) increased to 50 mL by adding distilled water), and 3. Triazine solution (47 mg triazine dissolved in 40 mL of 40 mM HCl). The working solution was prepared by adding 10 mL of the first solution to 1 mL of the second solution and 1 mL of the third solution. Furthermore, 25 µL of the serum sample and 25 µL of the homogenized brain sample were added to 1.5 mL of the working solution and stored at 37 °C. Optical absorbance was measured at 593 nm (Benzie and Strain 1999).

### Measuring serum and brain MDA levels

To measure the serum MDA level, 0.5 g of thiobarbituric acid was mixed with 80 mL of 20% acetic acid and then, the PH was adjusted to 3.5 using NaOH and its volume was increased to 100 mL by adding 20% acetic acid. Also, 100 µL of the serum sample was mixed with 100 µL of 8.1% SDS and 2.5 mL of the working solution. The samples were left in a bain-marie for 60 min and cooled, and then centrifuged at 4000 rpm. The optical absorbance of the supernatant was recorded at 523 nm wavelength. In order to measure the brain MDA level, 1 g of the brain tissue was dissolved in cooled 2.5% KCL and the resulting solution was homogenized at 10% w/v ratio, and then it was incubated at 37 ± 1 °C for 60 min in a metabolic shaker. Then, 1 mL of 5% tetrachloroacetic acid and 1 mL of 67% thiobarbituric acid were added to the solution and it was perfectly mixed after each addition. The content of each vial was centrifuged at 2000 rpm for 15 min. Then, the supernatant was transferred to another tube and left in a bain-marie. 10 min later, the tubes were cooled and the optical absorbance was recorded at 535 nm (Karatas et al. 2002).

### Statistical analysis

Data were analyzed in the GraphPad Prism software using one-way ANOVA followed by Tukey’s *post hoc* test. The significance level (*p*) was considered <0.05 in all calculations.

## Results

### Total phenolic and flavonoid contents and antioxidant capacity of *Ruta graveolens* extract

The amounts of phenolic and flavonoid compounds in the *R*. *graveolens* extract were 14.1 ± 0.47 mg GAE/g of the dry extracts and 15.8 ± 0.19 mg rutin equivalent/g of the dry extract, respectively. The obtained inhibitory activity of the *R*. *graveolens* extract in DPPH radicals (IC_50_) was 159.17 ± 1.56 μg/mL.

### Standardisation and determination of flavonoids and phenolic compounds by HPLC

The flavonoids and phenolic compounds, found in the herb extract, were detected by comparing the inhibition time of the samples’ peak levels with flavonoids and phenolic compounds standards, and the amounts of the compounds were calculated through calculating the sub-peak level. Five flavonoids (apigenin, luteolin, naringenin, quercetin, and rutin) and one phenolic acid (caffeic acid) were identified and their amounts were measured by the lowest and highest inhibition time obtained for caffeic acid and quercetin, respectively (Table 1).

**Table 1.** Flavonoids and phenolic compounds (mg/g of dry extract).

Caffeic acid	Rutin	Quercetin	Naringenin	Luteolin	Apigenin
19.92 ± 0.01	40.15 ± 0.01	ND	ND	ND	0.84 ± 0.01

ND: Non-detectable.

### The effect of *Ruta graveolens* extract and rutin on learning and spatial memory in Morris Water Maze test

There were significant differences in two-way ANOVA between different groups (*p* < 0.001). Bonferroni post hoc test showed that, latency to find the platform on day 3 was significantly lower in the groups receiving the extract with concentrations of 100 and 300 mg/kg and the group treated with rutin (10 mg/kg), compared to the control group (*p* < 0.05, *p* < 0.01, *p* < 0.001, respectively), but there was no significant difference between the group receiving the extract with the concentration of 30 mg/kg and the control group (*p* > 0.05). There was also no significant difference in latency between the groups receiving 100 and 300 mg/kg of the extract and the group receiving rutin, but there was a statistically significant difference between the group receiving the extract with concentration of 30 mg/kg and the group receiving rutin (*p* < 0.01). There was also a significant difference between two of the extract groups (30 and 300 mg/kg) (*p* < 0.05).

The latency in finding the platform on day 4 was significantly lower in the groups receiving 100 and 300 mg/kg of the extract and the group treated with rutin (10 mg/kg) compared to the control (*p* < 0.001), but there was no significant difference between the group receiving 30 mg/kg of the extract and the control group (*p* > 0.05). There was also no significant difference in latency between two of the extract (100 and 300 mg/kg) groups and the group receiving rutin, but there was a statistically significant difference between the group receiving the extract with the concentration of 30 mg/kg and the group receiving rutin (*p* < 0.001). There was also a significant difference between two of the extract groups (30 and 300 mg/kg) (*p* < 0.01).

**Figure 1.**
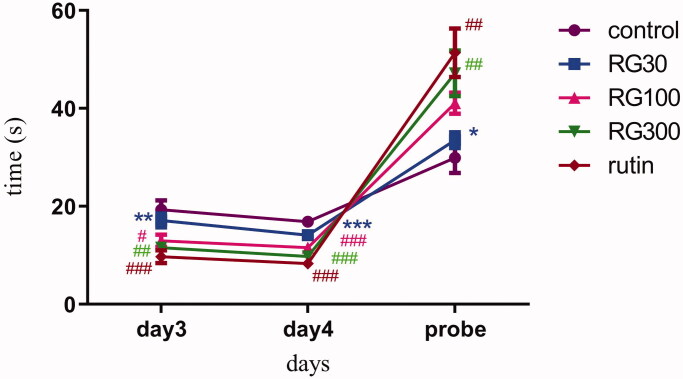
Effect of intraperitoneal injection of *Ruta graveolens* L. extract and rutin on latency to find the platform on day 3, 4 and duration of swimming in the tank ring target quadrant; RG30, RG100 and RG300: groups treated with 30, 100 and 300 mg/kg of *R. graveolens* extract; #, ##, ### significant difference between control group and other groups (*p* < 0.05, *p* < 0.01, and *p* < 0.001), **significant difference between rutin group and extract (*p* < 0.01) on day 3. ###significant difference between control group and other groups (p < 0.001), ***significant difference between rutin group and extract (*p* < 0.001) on day 4. ##significant difference between control group and other groups (*p* < 0.01), *significant difference between rutin group and extract (*p* < 0.05) on probe day.

The swimming duration in the target quadrant on the probe day, was significantly higher in the group receiving 300 mg/kg of the extract and the group treated with rutin (10 mg/kg) compared to the control group (*p* < 0.01), but there was no significant difference between the groups treated with 30 and 100 mg/kg of the extract and the control group (*p* > 0.05). There was also no significant difference in the swimming duration between two of the extract groups (100 and 300 mg/kg) and the group receiving rutin, but there was a statistically significant difference between the other extract group (30 mg/kg) and the group receiving rutin (*p* < 0.05). There was also a significant difference between two of the extract groups (30 and 300 mg/kg) (*p* < 0.05) (Figure 1).

### The effect of *Ruta graveolens* extract and rutin on avoidance memory in shuttle box test

**Figure 2.**
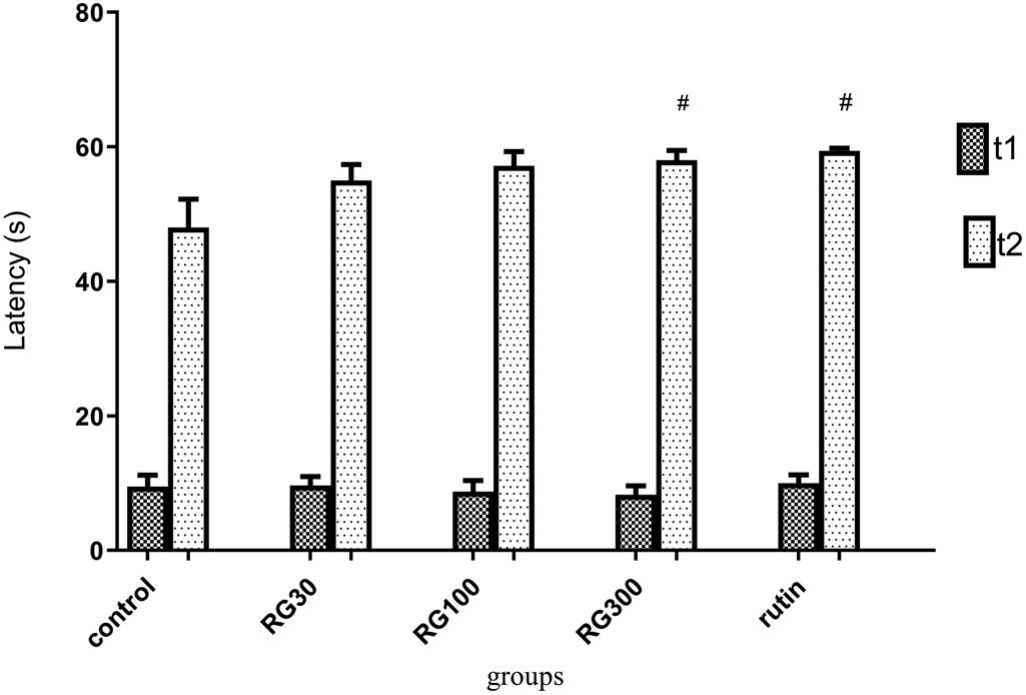
Effect of intraperitoneal injection of *Ruta graveolens* L. extract and rutin on passive avoidance memory; RG30, RG100 and RG300: groups treated with 30, 100 and 300 mg/kg of *R. graveolens* extract; # significant difference between control group and other groups (*p* < 0.05).

There were significant differences between different groups in one-way ANOVA (*p* < 0.001). Tukey’s *post hoc* test showed that secondary latency was significantly increased in the groups treated with 300 mg/kg of the extract and rutin (10 mg/kg) compared to the control group (*p* < 0.05), but this increase was not statistically significant in the groups treated with 30 and 100 mg/kg of the extract (*p* > 0.05). Also, there was no significant difference in secondary latency between the groups receiving the extract (30, 100, and 300 mg/kg) and the group receiving rutin (*p* > 0.05). There was also no significant difference between the groups receiving the extract (30, 100, and 300 mg/kg) (*p* < 0.05) (Figure 2).

### The effect of *Ruta graveolens* extract and rutin on serum antioxidant capacity

**Figure 3.**
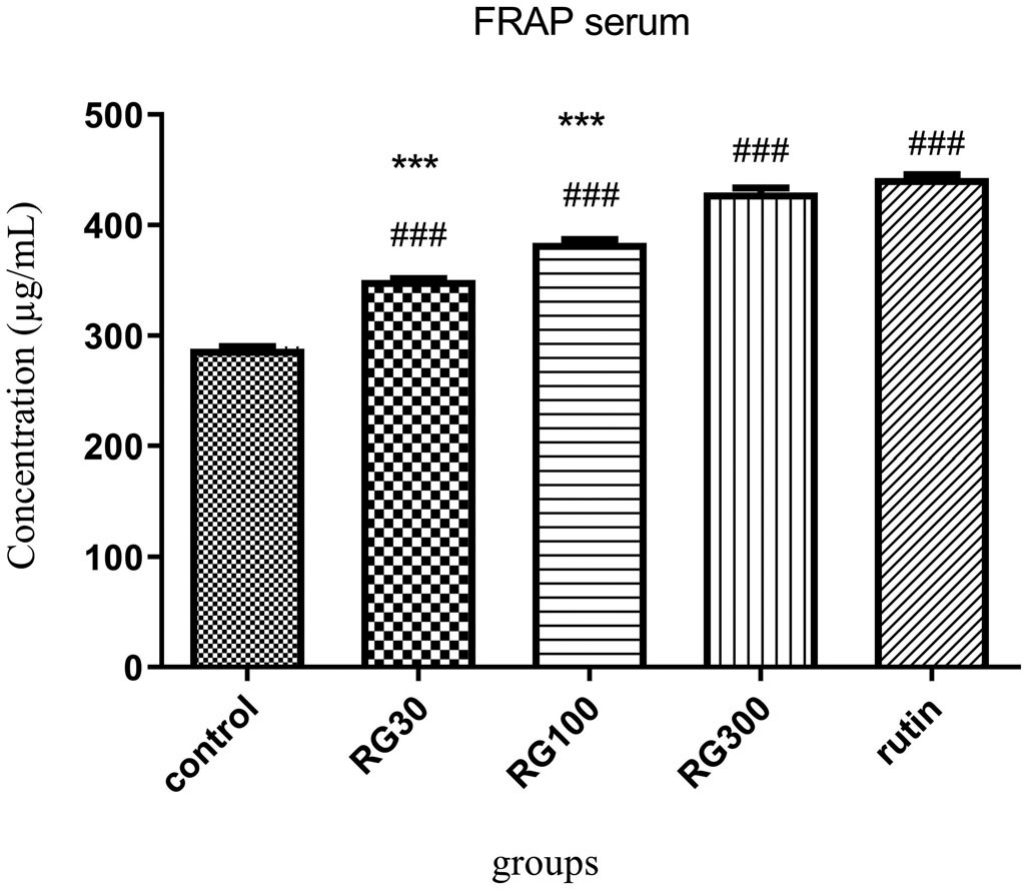
Effect of intraperitoneal injection of *Ruta graveolens* L. extract and rutin on serum antioxidant capacity; RG30, RG100 and RG300: groups treated with 30, 100 and 300 mg/kg of *R. graveolens* extract; ### significant difference between control group and other groups (*p* < 0.001), *** significant difference between rutin group and extract (*p* < 0.001).

There were significant differences between different groups in one-way ANOVA (*p* < 0.001). Tukey’s *post hoc* test showed that in the groups receiving 30, 100 and 300 mg/kg of the extract and the group receiving rutin (10 mg/kg), serum antioxidant capacity was significantly increased, compared to the control group (*p* < 0.001). According to the serum antioxidant capacity, there was no significant difference between one of the groups receiving extract (300 mg/kg) and the group receiving rutin (*p* > 0.05), but the difference between two of the groups receiving extract (30 and 100 mg/kg) and rutin-receiving group was significant (*p* < 0.001). There was also a significant difference between the groups receiving extract (30, 100, and 300 mg/kg) (*p* < 0.001) (Figure 3).

### The effect of *Ruta graveolens* extract and rutin on brain antioxidant capacity

**Figure 4.**
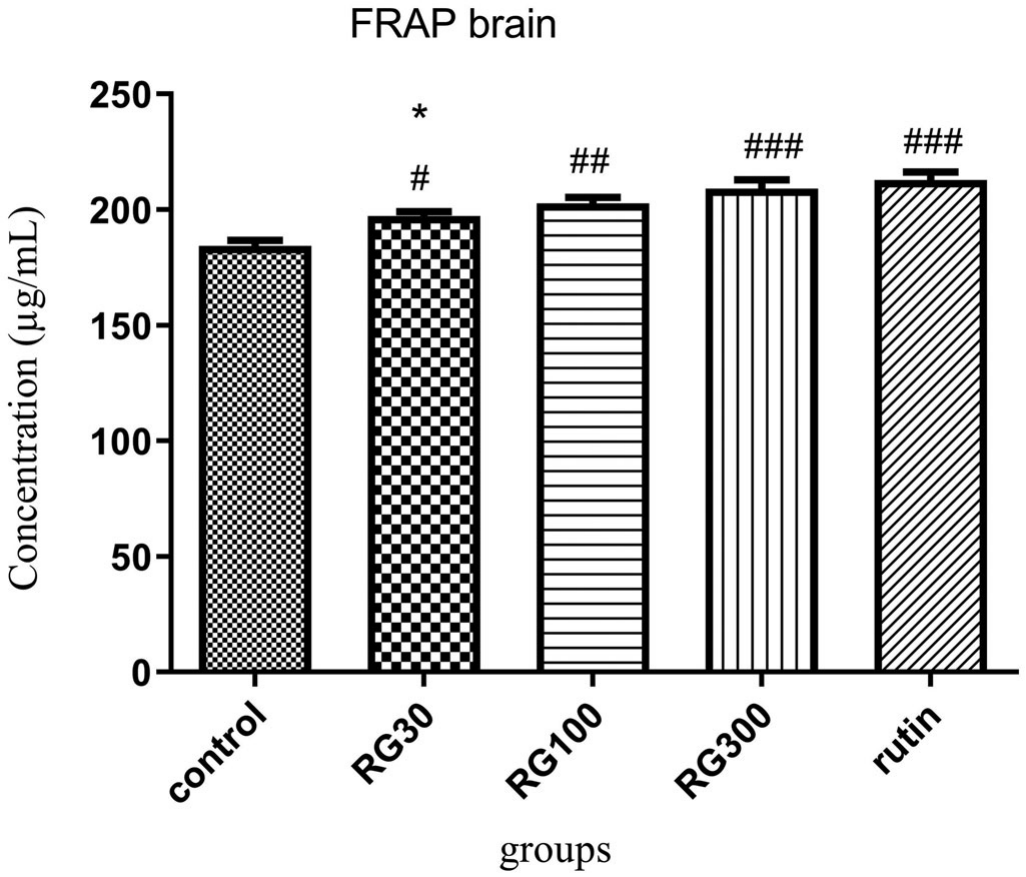
Effect of intraperitoneal injection of *Ruta graveolens* L. extract and rutin on brain antioxidant capacity; RG30, RG100 and RG300: groups treated with 30, 100 and 300 mg/kg of *R. graveolens* extract; #, ##, ### significant difference between control group and other groups (*p* < 0.05, *p* < 0.01, and *p* < 0.001), *significant difference between rutin group and extract (*p* < 0.05).

There were significant differences between different groups in one-way ANOVA (*p* < 0.001). Tukey’s *post hoc* test showed that the brain antioxidant capacity was significantly higher in the groups receiving *Ruta graveolens* extract with the concentrations of 30, 100, and 300 mg/kg and the group receiving 10 mg/kg rutin, compared to the control group (*p* < 0.05, *p* < 0.01, and *p* < 0.001, respectively). Regarding the brain antioxidant capacity, there was no significant difference between two of the groups receiving extract (100 and 300 mg/kg) and the group receiving rutin (*p* > 0.05), but the difference between one of the groups receiving extract (30 mg/kg) and the group receiving rutin was significant (*p* < 0.05). There was also a significant difference between two of the groups receiving extract (30 and 300 mg/kg) (*p* < 0.05) (Figure 4).

### The effect of *Ruta graveolens* extract and rutin on serum MDA level

**Figure 5.**
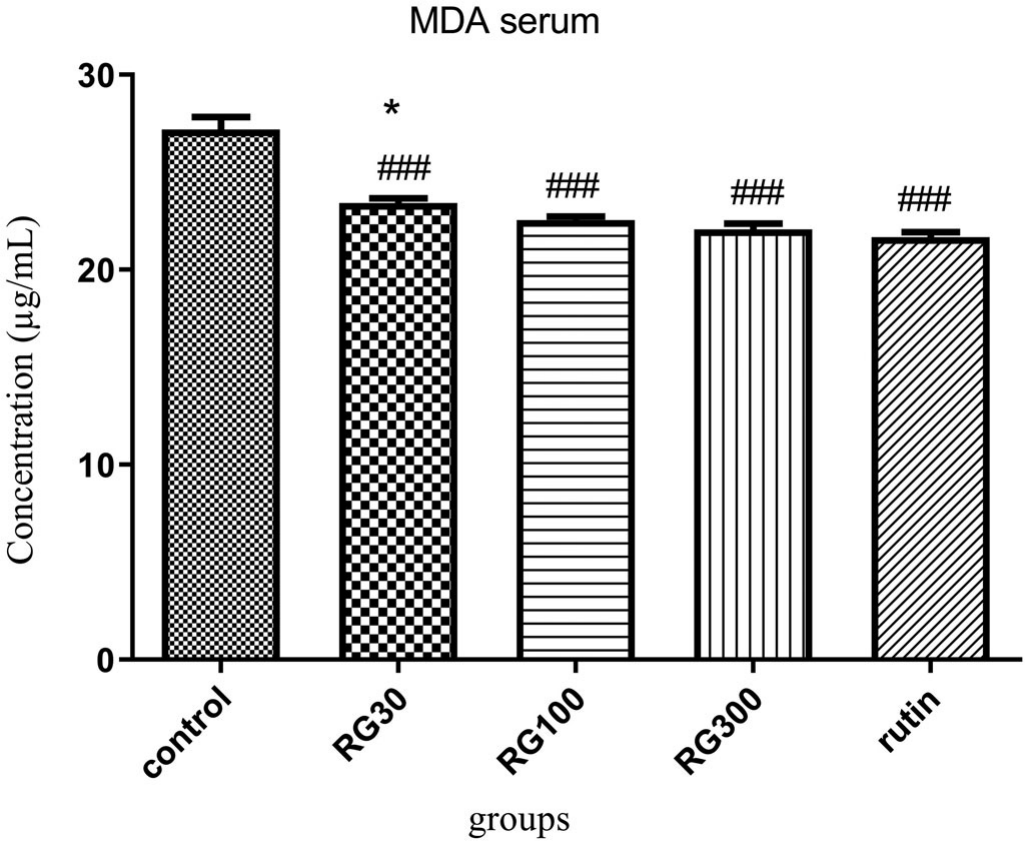
Effect of intraperitoneal injection of *Ruta graveolens* L. extract and rutin on serum malondialdehyde level; RG30, RG100 and RG300: groups treated with 30, 100 and 300 mg/kg of *R. graveolens* extract; ###significant difference between control group and other groups (*p* < 0.001), *significant difference between rutin group and extract (*p* < 0.05).

There were significant differences between different groups in one-way ANOVA (*p* < 0.001). Tukey’s post hoc test showed that serum MDA level was significantly lower in the groups receiving *R*. *graveolens* extract with the concentrations of 30, 100, and 300 mg/kg and the group receiving rutin (10 mg/kg), compared to the control group (*p* < 0.001). There was no significant difference in serum MDA level between two of the groups receiving extract (100 and 300 mg/kg) and the group receiving rutin (*p* > 0.05), but the difference between one of the groups receiving extract (30 mg/kg) and the group receiving rutin was significant (*p* < 0.05). There was also a significant difference between two of the groups receiving extract (30 and 300 mg/kg) (*p* < 0.05) (Figure 5).

### The effect of *Ruta graveolens* extract and rutin on brain MDA level

**Figure 6.**
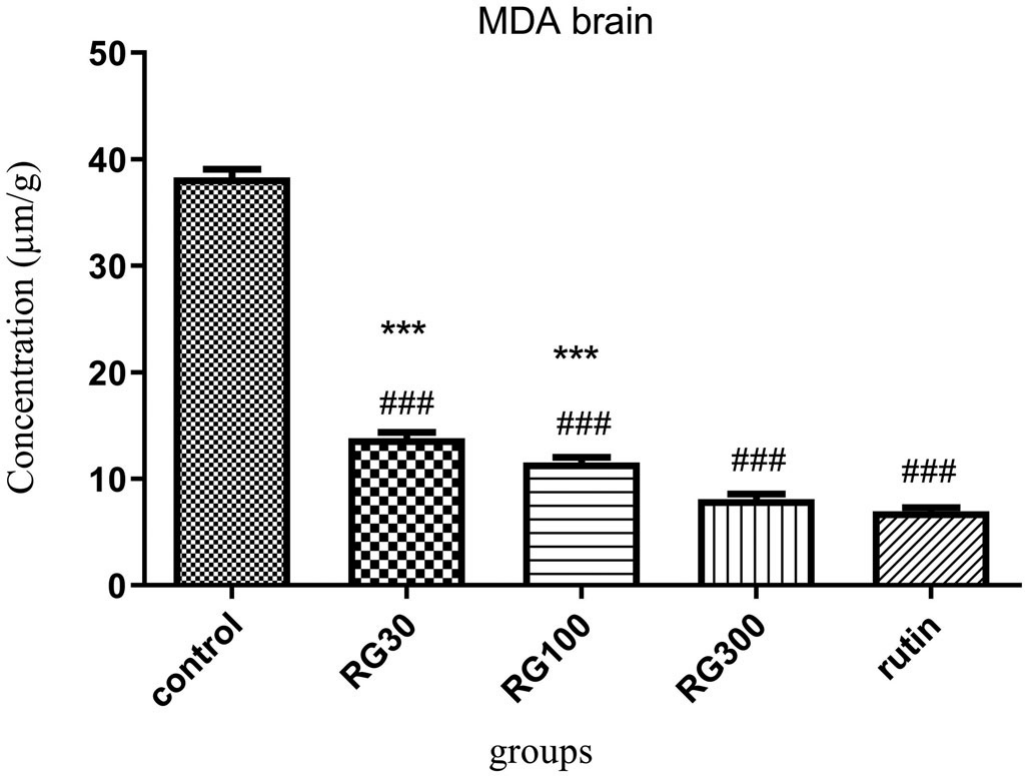
Effect of of *Ruta graveolens* L. extract and rutin on brain malondialdehyde level; RG30, RG100 and RG300: groups treated with 30, 100 and 300 mg/kg of *R. graveolens* extract; ###significant difference between control group and other groups (*p* < 0.001), ***significant difference between rutin group and extract (*p* < 0.001).

There were significant differences between different groups in one-way ANOVA (*p* < 0.001). Tukey’s *post* test showed that the brain MDA level was significantly decreased in the groups receiving *R*. *graveolens* extract with the concentrations of 30, 100, and 300 mg/kg and the group treated with rutin **(**10 mg/kg**),** compared to the control group (*p* < 0.001). There was no significant difference in brain MDA between the group receiving 300 mg/kg of the extract and the group receiving rutin (*p* > 0.05), but the difference between two of the groups receiving extract (30 and 100 mg/kg) and the group receiving rutin was significant (*p* < 0.001). There were also significant differences between the groups receiving extract (30 and 100 mg/kg, 30 and 300 mg/kg, and 100 and 300 mg/kg) (*p* < 0.05). The results of this test showed that the effects of this extract were dose-dependent (Figure 6).

## Discussion

Poor memory, low memory retention, and slow recall are the common problems in a world full of competition and stress. Aging, stress, and emotions are some of the leading cause of memory loss, forgetfulness, anxiety, high blood pressure, dementia, or even more serious complications, such as Alzheimer and schizophrenia. For centuries, natural compounds have been used by human beings for their health and well-being. Recently, demands for natural compounds and drugs have been increased due to the numerous side effects and the cost of these synthetic drugs used for treating disorders of the nervous system, learning, and memory (Forouzanfar and Hosseinzadeh 2018; Safiaghdam et al. 2018).

The group receiving 300 mg/kg of the extract and the group receiving rutin, compared to the control group, which this indicates the improving effect of *R*. *graveolens* on the spatial memory. There was a significant difference between the doses 30 and 300 mg/kg in comparing Morris water maze test results in the groups receiving extract. There was also a significant difference in the test results between one of the groups receiving extract (30 mg/kg) and the group receiving rutin. In addition, according to the shuttle box test results, there was no significant difference between different doses of extract and rutin. The results of the current study indicated that total serum and brain antioxidant capacity was significantly higher in the groups receiving 30, 100, and 300 mg/kg of the extract and the group receiving rutin, compared to the control group. There were also significant differences in serum antioxidant capacity between the groups receiving extract (30, 100 and 300 mg/kg), and there was another one in brain antioxidant capacity between two of the groups receiving extract (30 and 300 mg/kg). There were also significant differences in serum antioxidant capacity between two of the groups receiving extract (30 and 300 mg/kg) and the group receiving rutin, and there was another one in brain antioxidant capacity between one of the groups receiving extract (30 mg/kg) and the group receiving rutin. In addition, serum and brain MDA levels were significantly decreased in the groups receiving 30, 100, and 300 mg/kg of the *R*. *graveolens* extract and the group receiving rutin, compared to the control group. There were also significant differences in serum MDA level between two of the groups receiving extract (30 and 300 mg/kg) and another one in brain MDA level between the groups receiving extract (30, 100, and 300 mg/kg). There was also a statistically significant difference in serum MDA level between one of the groups receiving extract (30 mg/kg) and the group receiving rutin, and there was another significant difference in brain MDA level between two of the groups receiving extract (30 and 100 mg/kg) and the group receiving rutin. In 2006, Adsersen et al. proved that the extract of *R. graveolens* can inhibit acetylcholinesterase activity. Therefore, this plant has been suggested to be effective in memory improvement (Adsersen et al. 2006). Talić et al. (2014) also investigated the effect of *R. graveolens* on the inhibition of acetylcholinesterase (AChE) and butyrylcholinesterase (BuChE), which are considered as promising strategies in the treatment of Alzheimer’s disease (AD). According to this study, the strongest inhibition effect on AChE was detected within water extract of rue (IC_50_ = 50 µg/mL) and there was also a significant inhibition effect on BuChE. These results are consistent with our results. In a study conducted by Ratheesh et al. (2011) it was reported that methanol extract of *R*. *graveolens* reduced the oxidative stress and inflammation, and improved pathology induced in aorta of hypercholesterolemic rats, such that the herb decreased the activities of cyclooxygenase-2 and myeloperoxidase, as well as the concentration of thiobarbituric acid reactive substance, and increased the antioxidant activities of certain enzymes such as glutathione, indicating the antioxidant activity of the herb, which this is in agreement with our study (Ratheesh et al. 2011). Preethi et al. (2006) also reported that the low (homeopathic) concentrations of *R*. *graveolens* had cytotoxic effects on human lymphoma and fibroblastoma cells by controlling the oxygen free radicals and preventing the peroxidation of membrane lipids, and also saved the life of animals suffering from cancer (Preethi et al. 2006). In the study of Abidin et al. (2004), implemented on the relationship between oxidative stress and memory, stressful periods and increased stress levels were associated with adverse effect on memory and memory retention (Abidin et al. 2004). Therefore, oxidative stress accelerates the aging process and behaviours related to depression, dementia, and impairments of memory and learning. MDA is a product of cell membrane lipids peroxidation. Increasing the amounts of these lipid peroxides in the nerve cells leads to cell damage. The body uses the antioxidant system to cope with oxidative substances. In addition to these systems, some medicinal herbs reduce the oxidative stress and its adverse outcomes due to the antioxidants (Pandya et al. 2013; Rabiei et al. 2014).

The role of secondary metabolites in medicinal herbs, especially flavonoids, has been demonstrated in producing antioxidant effects in treating diseases related to oxidative stress, such as memory disorders and degenerative and inflammatory diseases (Bahmani et al. 2015; Kazemi et al. 2018). Rutin is one of the most important flavonoids, which is effective in memory impairment through its antioxidative activity (Ramalingayya et al. 2017). *R*. *graveolens* is one of the medicinal herbs that has high amounts of flavonoids, especially rutin, and phenolic acids, particularly caffeic acid. The current study separately evaluated and confirmed the positive effects of rutin on memory and learning.

In a study by Raghav et al. (2006), implemented on the anti-inflammatory effects of *R*. *graveolens* lavender extract on adipose tissue macrophages, it was indicated that the herb has an active flavonoid metabolite, which a large part of this flavonoid metabolite is consisted of rutin (Raghav et al. 2006).

In standardising the extract in the present study, the amounts of rutin, apigenin, and caffeic acid were 40.15, 0.84, and 19.92 μg/mL of dry extract, respectively, using HPLC analysis. In a study by Proestos et al. (2006), the amounts of phenolic and flavonoid compounds were measured in aromatic plants, such as *R*. *graveolens*, in which the amounts of caffeic acid, rutin, and quercetin were 20.1, 40.1 and 3.1 μg/mL of dry extract, respectively, and the amounts of apigenin, luteolin, and naringenin were not adequate to be detected. Besides that, the antioxidant capacity of the *R*. *graveolens* leaf was 159.17 μg/mL in the DPPH method (IC_50_), and the total phenolic content of the herb extract was calculated at 14.1 mg/g of dry extract.

These results ​​indicate the presence of antioxidant substances in the herb, most important of which are rutin and caffeic acid. The investigations of Fernandes et al. (2014) showed that caffeic acid could improve the memory loss in mice through cholinergic mediators and by coping with oxidative stress.

The beneficial effects of this herb on memory and learning are likely to be due to the presence of caffeic acid and its efficacy is through this pathway. In addition, as the previous studies have demonstrated the effect of rutin in reducing oxidative stress and its optimal effects on memory (Habtemariam 2016), the results of this part of our study confirms the presence of high amounts of rutin in the herb. The properties of rutin observed in different tests in our study seem to be due to its antioxidant properties and the resulting decrease in inflammatory substances that damage the neurons.

The inhibitory effects of rutin on GABA transaminase (GABA-T) and succinate-semialdehyde dehydrogenase have been indicated in numerous in-vitro studies. Inhibition of the two enzymes in the brain tissue increases GABA levels and may have therapeutic applications in neurological disorders. In addition, GABA-T is known as an important target for some of the drugs effective in the brain. Therefore, the inhibition of flavonoids by these enzymes may lead to exhibiting neuro-pharmacological effects (Tao et al. 2008). Therefore, it seems that improving memory and learning in the group receiving rutin in our study may also be due to increased amount of GABA.

In general, the memory-enhancing effects of the extract were increased by increasing the dose of the *R*. *graveolens* extract, so that the memory-enhancing-effect of the 300 mg/kg of the extract and rutin is stronger than 30 and 100 mg/kg of the extract. By comparing the results of total extract and the pure substance of rutin as the main substance of this plant, it can be concluded that rutin is sufficiently potent in comparison to the 30 and 100 mg/kg of the extract. In most tests, the extract had relatively similar strength to rutin (10 mg/kg) only at 300 mg/kg. According to the results of the HPLC analysis, it seems that about 249 mg of the extract contains 10 mg of rutin, which confirms the equivalent response of rutin to that of the extract at the dose of 300 mg/kg. However, the effect of the extract at this dose can also be attributed to a phenolic acid, namely, caffeic acid, in addition to its main compound rutin.

## Conclusion

The results of this study showed that intraperitoneal injection of hydroalcholic extract of *R*. *graveolens* and rutin, due to their potent antioxidant activities, increased serum and brain antioxidant capacity and decreased serum and brain MDA levels. This herb is likely to enhance memory and learning performance through these mechanisms. It is therefore suggested that after additional studies on animal samples and human subjects, the herb can be used as a supplement in preventing and reducing the adverse effects of oxidative stress, regarding the acceleration of the aging process and behaviours associated with depression, dementia, and memory and learning disorders.
